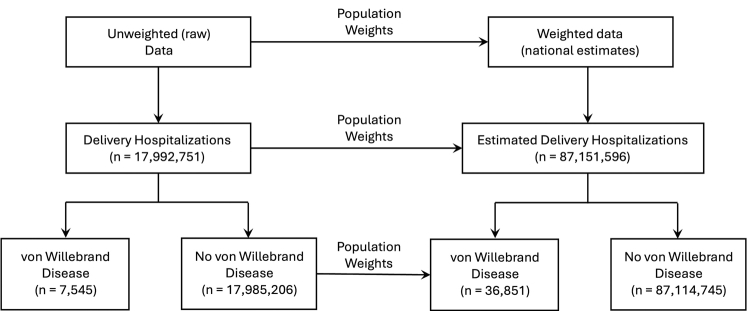# Contemporary trends in maternal outcomes during delivery hospitalizations among pregnancies complicated by von Willebrand disease—a cross-sectional analysis

**DOI:** 10.1016/j.rpth.2025.103174

**Published:** 2025-09-05

**Authors:** Minhazur R. Sarker, Rachel Wiley, Vishesh Khanna, Alexander M. Friedman, Timothy Wen

**Affiliations:** 1 Division of Maternal–Fetal Medicine, Department of Obstetrics, Gynecology and Reproductive Science, University of California San Diego, San Diego, California, USA; 2 Division of Hematology and Medical Oncology, Knight Cancer Institute, Oregon Health and Science University, Portland, Oregon, USA; 3 Division of Maternal–Fetal Medicine, Department of Obstetrics and Gynecology, Columbia University Irving Medical Center, New York, New York, USA; 4 Division of Biomedical Informatics, Department of Medicine, University of California San Diego, San Diego, California, USA

**Keywords:** hemorrhage, inherited bleeding disorder, severe maternal morbidity, transfusion, von Willebrand disease

## Abstract

**Background:**

While guideline-based multidisciplinary care is increasingly emphasized for managing von Willebrand disease (VWD) in pregnancy, most outcomes data are derived from outdated studies.

**Objectives:**

This study aimed to evaluate temporal trends in the prevalence of VWD, estimate hemorrhagic complication trends with VWD, and examine associations with adverse pregnancy outcomes with VWD.

**Methods:**

We conducted a cross-sectional analysis leveraging data from the National Inpatient Sample from 2000 to 2022 and identified VWD delivery hospitalizations using International Classification of Diseases codes. Outcomes included placental abruption or antepartum hemorrhage, postpartum hemorrhage, transfusion, nontransfusion severe maternal morbidity, and cesarean and operative vaginal delivery. Joinpoint regression was used to analyze trends by estimating the average annual percentage change. Unadjusted and adjusted logistic regression models were used to determine the strength of association between VWD and adverse pregnancy outcomes.

**Results:**

Among 87,151,596 delivery hospitalizations, 4.2 per 10,000 had a diagnosis of VWD. VWD prevalence rose from 2.1 to 5.1 per 10,000 deliveries between 2000 and 2022 (average annual percentage change, 6.6%; 95% CI, 5.3%-19.5%). Delivery hospitalizations with VWD were associated with increased rates of antepartum hemorrhage, postpartum hemorrhage, transfusion, nontransfusion severe maternal morbidity, and cesarean delivery. Of these associations, during the study period for deliveries with VWD, rates of antepartum hemorrhage and transfusion decreased significantly, and delivery route showed a decrease in operative vaginal delivery.

**Conclusion:**

Declining transfusion and antepartum hemorrhage rates suggest improvements in diagnosis and management of VWD during pregnancy. However, stable rates of postpartum hemorrhage rate highlight continued gaps in care. These contemporary, population-level findings will inform preconception counseling and intrapartum planning for individuals with VWD.

## Essentials

• Contemporary trends/outcomes for von Willebrand disease in pregnancy are poorly defined.
• Cross-sectional analysis was performed using United States National Inpatient Sample from 2000 to 2022.
• Prevalence of von Willebrand disease increased from 2.1 to 5.1 per 10,000 delivery hospitalizations.
• Temporal declines were noted in rates of transfusion and placental abruption/antepartum hemorrhage.

## 1 Introduction

Inherited bleeding disorders are a significant risk factor for hemorrhage during pregnancy, a primary cause of maternal morbidity and mortality [1,2]. von Willebrand disease (VWD), the most common inherited bleeding disorder, is characterized by reduced or dysfunctional von Willebrand factor and is associated with an increased risk of obstetric bleeding, including postpartum hemorrhage [3]. Advances in diagnosis and multidisciplinary care have enabled more individuals with VWD to safely carry pregnancies and subsequently, deliver newborns [4]. Furthermore, clinical care pathways for VWD in pregnancy have been designed by the American College of Obstetricians and Gynecologists and the Society for Maternal–Fetal Medicine in conjunction with American Society of Hematology to decrease the risk of hemorrhagic complications by leveraging multidisciplinary team based management, monitoring of coagulation status, prophylactic tranexamic acid after delivery, and postpartum tranexamic acid after hospital discharge [[3], [4], [5], [6], [7]].

Despite VWD being the most prevalent inherited bleeding disorder during pregnancy, contemporary data on associated adverse outcomes remains limited [3]. Currently, for pregnancies with VWD, adverse outcome profiles are determined primarily by prior population-level cross-sectional analysis from the 2000-2003 Nationwide Inpatient Sample noting increased hemorrhagic complication, with subsequent literature largely limited to small case series or survey-based studies [8]. The real-world impact of the multidisciplinary care models and clinical management guidelines that have been introduced to optimize outcomes in this high-risk patient population remains unclear [5].

To address this gap, we conducted a contemporary cross-sectional analysis using national data to (1) evaluate temporal trends in VWD prevalence among hospitalizations for delivery, (2) assess year-over-year changes in the prevalence of common hemorrhagic complications of pregnancy among deliveries with a VWD diagnosis, and (3) to determine associations between VWD and adverse pregnancy outcomes. We hypothesized that with guideline recommendations diffusing into practice, the prevalence of VWD among delivery hospitalizations would increase over time, accompanied by a corresponding decrease in hemorrhagic complications and other adverse obstetric outcomes.

## 2 Methods

This is a cross-sectional analysis leveraging data from the Healthcare Cost and Utilization Project’s (HCUP) National Inpatient Sample (NIS) [9]. The NIS is one of the largest publicly available, all payer, inpatient databases in the United States and approximates a 20% stratified sample of all hospitalizations annually. In 2022, data from 47 states and the District of Columbia were included in the NIS [10]. Annually, more than 7 million hospital stays are included in the unweighted NIS sample; the data can be weighted to create national estimates of 35 million hospital stays by applying NIS sample weights. This study includes the transition period from International Classification of Diseases, Ninth Revision, Clinical Modification (ICD-9-CM) to the Tenth Revision (ICD-10-CM). Given that, data from the NIS included both ICD-9-CM and ICD-10-CM codes, which required translation using an algorithm using publicly available General Equivalence Mappings provided by the Centers for Medicare & Medicaid Services and National Center for Health Statistics [11,12].

Delivery hospitalizations to persons’ age 15 to 54 years were identified using diagnosis and procedure ICD-9CM and ICD-10CM codes in NIS between 2000 and 2022 using previously published methods (Supplementary Table 1) [13]. Hospitalizations with VWD were identified by ICD-9-CM code 286.4 and ICD-10-CM code D68.0. Given limitations of ICD coding, we are unable to distinguish between VWD subtypes.

This study has 3 main objectives: (1) evaluate temporal trends in the VWD prevalence during delivery hospitalizations, (2) estimate temporal trends of common hemorrhagic complications of pregnancy among deliveries with a VWD diagnosis, and (3) determine associations between VWD diagnosis and adverse pregnancy outcomes. Using ICD-9-CM and ICD-10-CM codes (Supplementary Table 1), adverse pregnancy outcomes were identified and included the following: hypertensive disorders of pregnancy, placental abruption or antepartum hemorrhage, postpartum hemorrhage, transfusion, thrombotic complications (a composite of acute myocardial infarction, stroke, deep venous thrombosis, and pulmonary embolism), nontransfusion severe maternal morbidity, cesarean delivery, operative vaginal delivery, stillbirth, and preterm birth stratified by less than 28, 32, or 37 weeks’ gestation. Severe maternal morbidity was defined based on composite measures as per the Centers for Disease Control and Prevention (CDC) [14]. The CDC composite includes 21 diagnoses and conditions including transfusion, shock, stroke, heart failure, sepsis, and all other conditions identified by ICD-9-CM and ICD-10-CM codes (Supplementary Table 1). This analysis excluded transfusion from the CDC composite and focused on the remaining 20 diagnoses and conditions. Transfusion was excluded from the composite and analyze separately given the clinical interest in assessing hemorrhagic complications.

Demographic, clinical factors, and hospital characteristics were evaluated based on the presence or absence of VWD. Demographic factors included maternal age stratified by categories (15-19, 20-24, 25-29, 30-34, 35-39, 40-44, 45-49, and 50-54 years), self-reported maternal race and ethnicity (non-Hispanic White, non-Hispanic Black, Hispanic, other, or unknown), payer status (Medicaid, Medicare, private, self-pay, other, or uninsured), median income quartile by zip code, and year of delivery. Race and ethnicity were included to assess disparities but were not presumed as intrinsically causal factors [[15], [16], [17]]. Clinical factors were identified using diagnosis codes and included pregestational diabetes, chronic hypertension, maternal obesity, asthma, prior cesarean delivery, and multifetal gestation. Hospital characteristics included teaching status (urban teaching, urban nonteaching, and rural) and geographic region (Northeast, Midwest, South, or West) [18].

For the first objective, evaluating trends in VWD prevalence over time, we used the weighted-sample to report the rate of delivery hospitalizations with VWD per 10,000 hospitalizations by year of delivery. We conducted trends analyzes using the National Cancer Institute’s Joinpoint Regression Program version 5.3.0 [19]. This program estimates the average annual percentage change (AAPC) over the whole study period and then allows the identification of when a trend change is produced and calculates the AAPC between trend change points. The AAPC is derived by estimating the underlying joinpoint model that best fits the data, using a weighted average of the slope coefficients of the underlying joinpoint regression line with the weights equal to the length of each segment, and finally, transforming the weighted average of slope coefficients. Data are reported as the AAPC with a 95% CI.

For the second objective, evaluating temporal trends of hemorrhagic complications and mode of delivery complications among delivery hospitalizations affected by VWD, we used the weighted-sample and the Joinpoint Regression Program to estimate AAPCs and 95% CIs specifically for placental abruption/antepartum hemorrhage, transfusion, postpartum hemorrhage, and operative vaginal deliveries.

For the third objective, evaluating the association between VWD and adverse pregnancy outcomes, we fit unadjusted and adjusted survey-weighted logistic regression models for each outcome comparing with and without VWD. Demographic analyses stratified the patient, clinical, and hospital features by presence or absence of VWD. The adjusted models were fitted adjusting for the above-mentioned demographic, hospital, and clinical factors to calculate the adjusted risk for delivery hospitalizations with VWD compared with those in hospitalizations without VWD. For the models evaluating hypertensive disorders of pregnancy, placental abruption or antepartum hemorrhage, postpartum hemorrhage, transfusion, thrombotic complications (a composite of acute myocardial infarction, stroke, deep venous thrombosis, and pulmonary embolism), nontransfusion severe maternal morbidity, cesarean delivery, and stillbirth, the cohort of analysis included all births meeting inclusion criteria from 2000 to 2022. For models evaluating operative vaginal delivery, the cohort of analysis was limited to vaginal births only meeting inclusion criteria from 2000 to 2022. For the models evaluating preterm birth outcomes, the cohort of analysis was limited to all births meeting inclusion criteria from 2016-2022 with nonmissing gestational age. Specific gestational age codes are only available in ICD-10-CM and were categorized using a hierarchal approach when multiple gestational age codes were present [20]. Associations are presented as unadjusted odds ratio and adjusted odds ratio (aOR) with 95% CI.

All analyses were performed with SAS 9.4 (SAS Institute) except for the temporal trend analyses performed with the Joinpoint Regression Program. We adhered to Strengthening the Reporting of Observational Studies in Epidemiology guidelines for cross-sectional studies for this analysis [21]. Given this study used deidentified publicly available data, the analysis was deemed exempt by the institutional review board.

## 3 Results

**Figure 1:**
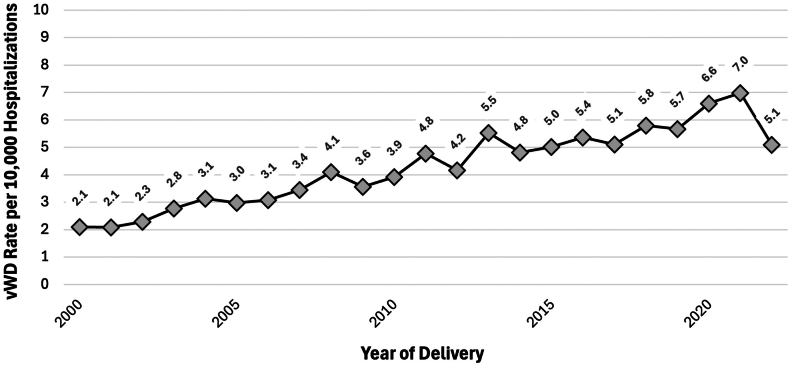
von Willebrand disease (VWD) prevalence per 10,000 delivery hospitalizations from 2000 to 2022 by year of delivery.

**Figure 2:**
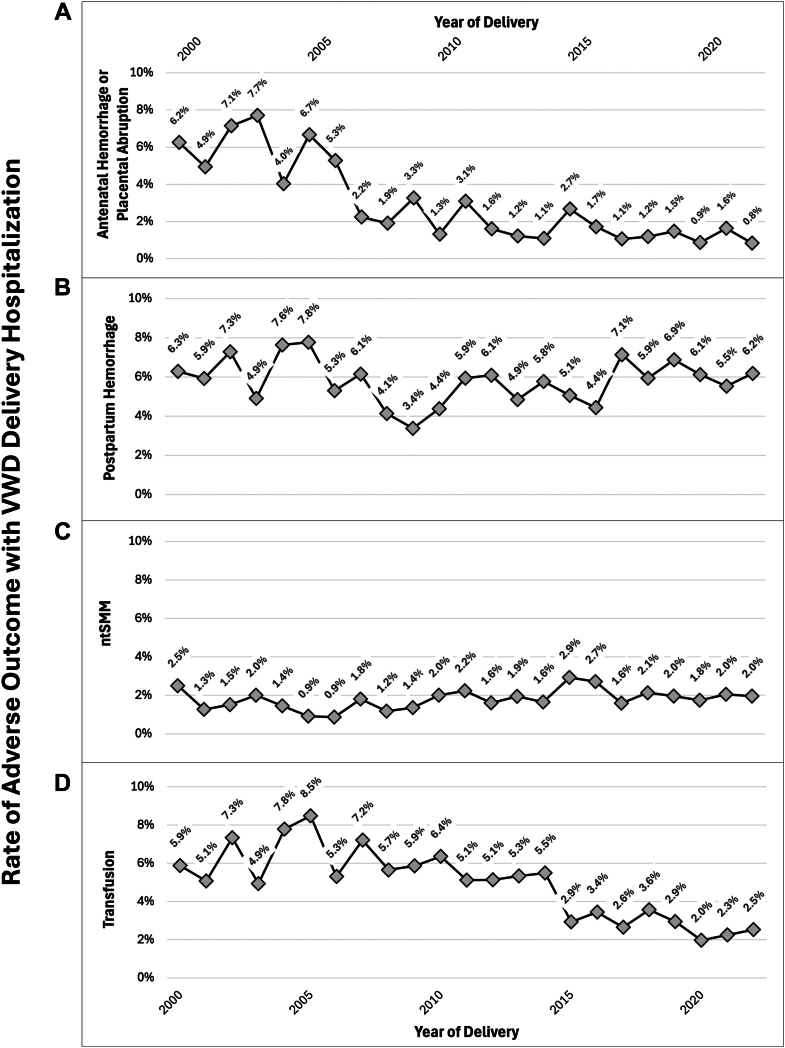
Rates of adverse outcomes among delivery hospitalizations with von Willebrand disease (VWD) from 2000 to 2022: (A) antenatal hemorrhage or placental abruption, (B) postpartum hemorrhage, (C) nontransfusion severe maternal morbidity (ntSMM), and (D) transfusion.

**Figure 3:**
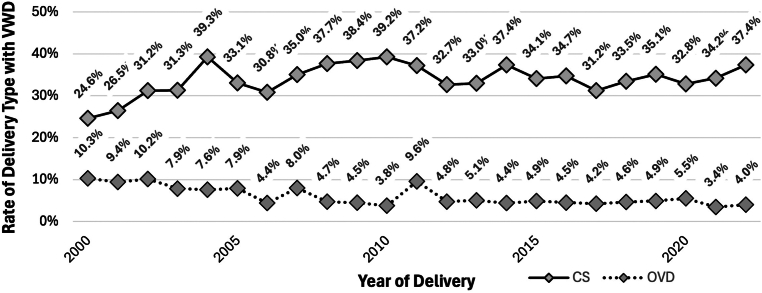
Mode of delivery trends from 2000 to 2022 among delivery hospitalizations with von Willebrand disease (VWD)—rate of cesarean delivery (CS) and operative vaginal delivery (OVD).

From 2000 to 2022 NIS, we identified 87,151,596 delivery hospitalizations, of which 4.2 per 10,000 were affected by VWD (*n* = 36,851) (Supplementary Figure). The prevalence of VWD among delivery hospitalizations increased from 2.1 to 5.1 cases per 10,000 delivery hospitalizations from 2000 to 2022 (AAPC, 6.6%; 95% CI, 5.3%-19.5%) (Figure 1). Among delivery hospitalizations with VWD from 2000 to 2022, there were significant declines in rates of placental abruption or antepartum hemorrhage (6.2% in 2000 to 0.8% in 2022; AAPC, -9.0%; 95% CI, -12.6% to -6.2%) and blood transfusion (5.9% in 2000 to 2.5% in 2022; AAPC, -5.2%; 95% CI, -7.3% to -3.3%) with no change in the rates of postpartum hemorrhage (6.3% in 2000 to 6.2% in 2022; AAPC, -0.1%; 95% CI, -1.6% to 1.8%) or nontransfusion severe maternal morbidity (2.5% in 2000 to 2.0% in 2022; AAPC, 1.4%; 95% CI, -0.4% to 3.9%) (Figure 2). Additionally, there was a significant decline in operative vaginal delivery rates from 10.3% in 2000 to 4.0% in 2022 (AAPC, -3.8%; 95% CI, -5.5% to -2.1%) and no change in cesarean delivery rates from 24.6% in 2000 to 37.4% in 2022 (AAPC, 0.2%; 95% CI, -0.5% to 1.1%) (Figure 3).

VWD was more common among deliveries to non-Hispanic White patients (1 in 1565) than that in non-Hispanic Black (1 in 4608) or Hispanic patients (1 in 4470) (Table 1). The delivery hospitalizations with VWD had higher prevalence of individuals who were privately insured and in the highest median income quartile (Table 1). Among clinical factors, delivery hospitalizations with VWD had higher prevalence of asthma, obesity, and chronic hypertension (Table 1). Among hospital factors, delivery hospitalizations with VWD had higher prevalence of deliveries occurring at urban teaching hospitals than those at urban nonteaching or rural hospitals (Table 1).

**Table 1:** Demographic, clinical, and hospital factors.

Covariate	von Willebrand disease	
	Absent (*n* = 87,114,745)	Present (*n* = 36,851)
Maternal age (y)		
15-19	6,965,331 (8.0)	2495 (6.8)
20-24	19,599,577 (22.5)	8489 (23.0)
25-29	24,338,017 (27.9)	10,984 (29.8)
30-34	22,463,895 (25.8)	9615 (26.1)
35-39	11,193,359 (12.8)	4303 (11.7)
40-54	2,554,565 (2.9)	965 (2.6)
Maternal race		
White	39,213,892 (45.0)	25,101 (68.1)
Black	10,420,137 (12.0)	2265 (6.2)
Hispanic	16,780,636 (19.3)	3755 (10.2)
Other	8,095,656 (9.3)	1761 (4.8)
Unknown	12,604,424 (14.5)	3968 (10.8)
Payer status		
Medicare	506,234 (0.6)	486 (1.3)
Medicaid	35,957,108 (41.3)	12,007 (32.6)
Private insurance	45,470,161 (52.2)	22,585 (61.3)
Self-pay	2,594,706 (3.0)	463 (1.3)
No charge	145,802 (0.2)	19 (0.1)
Other	2,303,533 (2.6)	1199 (3.2)
Missing	137,202 (0.2)	92 (0.2)
Median income quartile by ZIP code		
Income quartile 1	21,183,658 (24.3)	7260 (19.7)
Income quartile 2	21,158,109 (24.3)	9024 (24.5)
Income quartile 3	21,264,561 (24.4)	9702 (26.3)
Income quartile 4	22,252,877 (25.5)	10,438 (28.3)
Missing	1,255,540 (1.4)	427 (1.2)
Clinical factors		
Chronic hypertension	1,174,468 (1.4)	827 (2.2)
Pregestational diabetes mellitus	871,399 (1.0)	313 (0.9)
Prior cesarean delivery	13,958,591 (16.0)	6061 (16.5)
Multiples gestation	1,568,876 (1.8)	897 (2.4)
Obesity	4,402,005 (5.1)	2541 (6.9)
Asthma	3,133,746 (3.6)	3199 (8.7)
Hospital teaching status		
Rural	9,369,636 (10.8)	2627 (7.1)
Urban nonteaching	29,767,986 (34.2)	9769 (26.5)
Urban teaching	47,749,776 (54.8)	24,370 (66.1)
Missing	227,346 (0.3)	85 (0.2)
Hospital region		
Northeast	14,100,000 (16.2)	10,112 (27.4)
Midwest	18,478,389 (21.2)	8594 (23.3)
South	33,282,228 (38.2)	11,018 (29.9)
West	21,254,128 (24.4)	7126 (19.3)
Delivery year		
2000	3,814,045 (4.4)	804 (2.2)
2001	3,749,008 (4.3)	785 (2.1)
2002	3,902,512 (4.5)	893 (2.4)
2003	3,860,991 (4.4)	1072 (2.9)
2004	3,997,744 (4.6)	1254 (3.4)
2005	4,008,146 (4.6)	1194 (3.2)
2006	4,059,251 (4.7)	1252 (3.4)
2007	4,327,048 (5.0)	1492 (4.1)
2008	4,010,396 (4.6)	1643 (4.5)
2009	3,915,670 (4.5)	1396 (3.8)
2010	3,683,708 (4.2)	1444 (3.9)
2011	3,645,450 (4.2)	1741 (4.7)
2012	3,748,116 (4.3)	1560 (4.2)
2013	3,725,963 (4.3)	2060 (5.6)
2014	3,782,605 (4.3)	1820 (4.9)
2015	3,736,190 (4.3)	1875 (5.1)
2016	3,780,456 (4.3)	2030 (5.5)
2017	3,700,941 (4.3)	1890 (5.1)
2018	3,633,239 (4.2)	2105 (5.7)
2019	3,586,949 (4.1)	2035 (5.5)
2020	3,457,561 (4.0)	2285 (6.2)
2021	3,492,747 (4.0)	2440 (6.6)
2022	3,496,010 (4.0)	1780 (4.8)

Values are *n* (%).

Delivery hospitalizations with VWD had higher rates of hypertensive disorders of pregnancy (11.0% vs 8.7%; OR, 1.29; 95% CI, 1.20-1.39), placental abruption or antepartum hemorrhage (2.5% vs 1.4%; OR, 1.83; 95% CI, 1.56-2.13), postpartum hemorrhage (5.7% vs 3.3%; OR, 1.79; 95% CI, 1.63, 1.98), blood transfusion (4.6% vs 1.0%; OR, 5.00; 95% CI, 4.46-5.60), and nontransfusion severe maternal morbidity (1.9% vs 0.7%; OR, 2.63; 95% CI, 2.22-3.12) (Table 2; Supplementary Table 2). VWD was associated with lower operative vaginal delivery rates (5.6% vs 7.8%; OR, 0.71; 95% CI, 0.63-0.80) and higher cesarean delivery rates (34.4% vs 31.1%; OR, 1.17; 95% CI, 1.11-1.23) (Table 2; Supplementary Table 2). There were no clinically meaningful differences noted between VWD and thrombotic complications, stillbirth, or preterm birth of any category (Table 2).

**Table 2:** Outcome demographics.

Outcome	von Willebrand disease	
	Absent, *n* (%)	Present, *n* (%)
Hypertensive disorders of pregnancy	7,608,000 (8.7)	4041 (11.0)
Placental abruption/antepartum hemorrhage	1,205,001 (1.4)	920 (2.5)
Postpartum hemorrhage	2,856,959 (3.3)	2113 (5.7)
Transfusion	824,128 (1.0)	1679 (4.6)
Thrombotic complication	81,903 (0.1)	60 (0.2)
Nontransfusion severe maternal morbidity	620,391 (0.7)	683 (1.9)
Cesarean delivery	27,061,673 (31.1)	12,661 (34.4)
Operative vaginal delivery	4,674,441 (7.8)	1358 (5.6)
Stillbirth	625,381 (0.7)	227 (0.6)
Preterm birth <28 wk	158,465 (0.6)	105 (0.7)
Preterm birth <32 wk	392,105 (1.6)	205 (1.4)
Preterm birth <37 wk	2,429,274 (9.9)	1545 (10.8)

**Figure 4:**
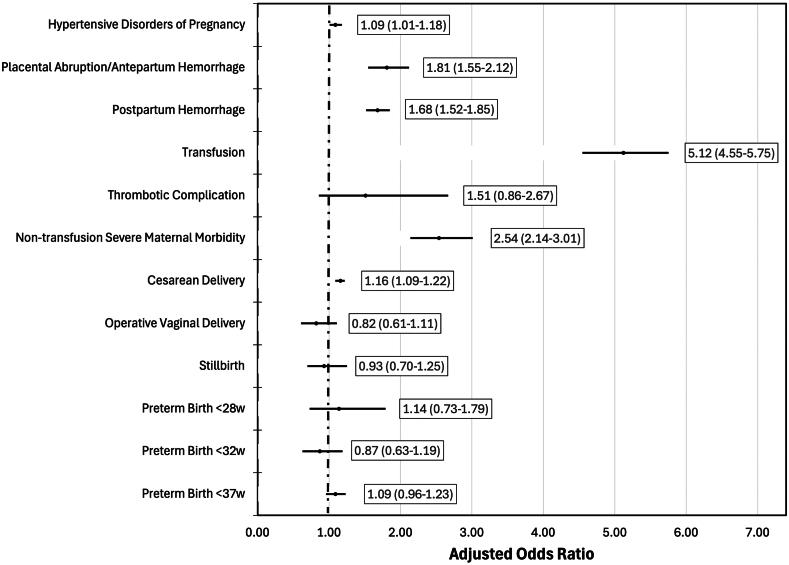
Multivariable logistic regression of outcomes associated with von Willebrand disease. The adjusted model included demographic factors (maternal age, maternal race, payer status, and median income quartile), clinical factors (chronic hypertension, pregestational diabetes, prior cesarean delivery, multifetal gestation, obesity, and asthma), hospital factors (teaching hospital status and region), and delivery year.

Adjusted analysis found that delivery hospitalizations with VWD were associated with higher likelihood of hypertensive disorders of pregnancy (aOR, 1.09; 95% CI, 1.01-1.18), placental abruption or antepartum hemorrhage (aOR, 1.81; 95% CI, 1.55-2.12), postpartum hemorrhage (aOR, 1.68; 95% CI, 1.52-1.85), transfusion (aOR, 5.12; 95% CI, 4.55-5.75), nontransfusion severe maternal morbidity (aOR, 2.52; 95% CI, 2.14-3.01), and cesarean delivery (aOR, 1.16; 95% CI, 1.09-1.22) (Figure 4). VWD was not associated with thrombotic complications, operative vaginal delivery, stillbirth, or preterm birth (Figure 4).

## 4 Discussion

In this contemporary nationally representative sample of delivery hospitalizations, our findings demonstrated an increasing prevalence of VWD over time, with decreasing rates of transfusion and placental abruption/antepartum hemorrhage among those births. There were no significant trends in postpartum hemorrhage or nontransfusion severe maternal morbidity. VWD continues to be associated with meaningfully higher odds of transfusion, antepartum and postpartum hemorrhage, and nontransfusion severe maternal morbidity. Our findings suggesting decreased proportions of operative vaginal delivery is likely a reflection of recent guidelines suggesting caution with operative vaginal delivery in patients with VWD [4]. Finally, our findings reveal an association between VWD and subsequent hypertensive disorder of pregnancy, which may be explained by the higher prevalence of baseline chronic hypertension and maternal obesity in our VWD study population.

The increasing prevalence of VWD among delivery hospitalizations and decreasing rates of hemorrhage-related complications is likely a reflection of improvements in identification and management of patients with VWD. Over the past 2 decades, multidisciplinary professional medical societies have developed clinical care pathways for managing these high-risk patients. This is supported by literature highlighting the importance of prepregnancy VWD diagnosis and multidisciplinary co-management by maternal–fetal medicine and hematology [[3], [4], [5]]. As postpartum hemorrhage rates in the general population are increasing, stable postpartum hemorrhage rates in our VWD population may suggest improvements in care. Our findings also note decreasing trends in other hemorrhagic complications such as placental abruption/antepartum hemorrhage and transfusion, suggesting temporal improvements in management of VWD. However, despite this improvement in hemorrhage-related outcomes, our findings note that pregnancies with VWD remain at elevated risk of adverse pregnancy outcomes, consistent with older studies [8,[22], [23], [24]]. Moreover, the increasing prevalence of VWD in pregnancy highlights the importance of having contemporary data on adverse outcomes to bolster management in a segment of the pregnant population with this rare high-risk condition.

Given the implementation of clinical care pathways, it is likely the increased prevalence is secondary to improved recognition and diagnosis rather than changes in population-level pathophysiology. Our findings indicated that among delivery hospitalizations, patients with VWD were disproportionately non-Hispanic White, privately insured, and from higher income zip codes. These patterns may reflect disparities in access to diagnostic evaluation and care, rather than true differences in disease prevalence. Moreover, it is possible that during the earlier study period, only more severe cases were being diagnosed, and over time, diagnosis of less severe variants increased. Thus, the decreased incidence of adverse outcomes may reflect differences in composition of VWD severity types. Owing to the limitations of the HCUP NIS, our analysis is unable to granularly comment on VWD subtypes. Nonetheless, since timely diagnosis is central to safe and effective peripartum management, efforts to improve recognition of bleeding symptoms in marginalized populations may be an important step in optimizing VWD management.

Our study has multiple limitations inherent to the use of administrative data. First, this study’s case and outcome ascertainment strategies were based on ICD-9-CM and ICD-10-CM codes, which have issues with misclassification. To our knowledge, no studies have directly assessed the validity of ICD-9 or ICD-10 codes for identifying VWD. Additionally, underdiagnosis is always a possibility clinically and thus would be underidentified in claims data. However, the rate of delivery hospitalizations with VWD is similar to that seen in our clinical experience. Although the prevalence of VWD in the unselected general population is estimated to be approximately 1%, cases with clinically significant burden are much more rare, which may explain the significantly smaller prevalence in our study [6]. Second, we are unable to comment on the type or amount of blood products that were transfused, or the interventions used, and resources present for management of postpartum hemorrhage in each case. Additionally, we are unable to comment on the granular management of VWD such as third trimester coagulation screening, prophylactic tranexamic acid after delivery, or postpartum tranexamic acid after hospital discharge. Third, using delivery hospitalizations as the unit of analysis does not allow us to adjust for multiple deliveries by the same patient. Fourth, cross-sectional analyses do not allow for causal inferences to be made. Fifth, we are unable to distinguish between VWD subtypes, which may have differing associations with pregnancy outcomes. For example, individuals with type 3 or type 2B VWD may have more severe bleeding phenotypes or require different peripartum management than those with type 1 VWD. Future research (eg, registries and prospective cohorts) should focus on examining differential outcomes in VWD subtypes. Sixth, transitioning midstudy from ICD-9-CM to ICD-10-CM with greater coding comprehensiveness might have resulted in some lapses, although our Joinpoint Regression did not identify trend point changes and we attempted to account for this transition by using the General Equivalence Mappings [11]. Finally, our data are limited to the United States, which decreases the generalizability of our findings.

Our study also has multiple strengths. First, HCUP NIS is the most reliable database for hospital admissions and discharges [10]. Second, we were able to leverage national estimates for this rare condition providing the ability to calculate nationally representative trends and detect small differences in clinical outcomes. Third, VWD, despite being most common bleeding disorder in pregnancy, remains relatively rare in the general population such that a detailed study, while providing granular data on VWD management, remains relatively unfeasible highlighting the importance of our analysis.

## 5 Conclusion

Our study found an increasing prevalence of VWD among delivery hospitalizations, with persistent associations between VWD and elevated obstetrical risks of hemorrhagic complications and nontransfusion–related severe morbidity. The observed declines in transfusions and antepartum hemorrhage may reflect the impact of guideline-based, multidisciplinary management. These contemporary, population-level estimates of VWD-related pregnancy risks can inform and improve preconception counseling, intrapartum planning, and postpartum care for pregnancies with VWD.

## Figures and Tables

**Supplementary Figure 1:**