# Supplementary material for: Contemporary trends in maternal outcomes during delivery hospitalizations among pregnancies complicated by von Willebrand disease—a cross-sectional analysis
Source: Res Pract Thromb Haemost. 2025 Sep 5;9(6):103174. doi: 10.1016/j.rpth.2025.103174 (PMC12509092; doi:10.1016/j.rpth.2025.103174)
Supplement: Supplementary Table 1 [file mmc1.docx]

| **Supplemental Table 1. Specific Codes used for Analyses** | | |
| --- | --- | --- |
| **Demographic, Clinical, or Hospital Factor** | **Type of Code** | |
|  | **ICD-9-CM** | **ICD-10-CM** |
| Von Willebrand Disease | 286.4 | D680 |
| Pregestational diabetes | 6480 , 64801, 64802, 64803, 64804, 249 , 2490 , 24900, 24901, 2491 , 24910, 24911, 2492 , 24920, 24921, 2493 , 24930, 24931, 2494 , 24940, 24941, 2495 , 24950, 24951, 2496 , 24960, 24961, 2497 , 24970, 24971, 2498 , 24980, 24981, 2499 , 24990, 24991, 250 , 2500 , 25000, 25001, 25002, 25003, 2501 , 25010, 25011, 25012, 25013, 2502 , 25020, 25021, 25022, 25023, 2503 , 25030, 25031, 25032, 25033, 2504 , 25040, 25041, 25042, 25043, 2505 , 25050, 25051, 25052, 25053, 2506 , 25060, 25061, 25062, 25063, 2507 , 25070, 25071, 25072, 25073, 2508 , 25080, 25081, 25082, 25083, 2509 , 25090, 25091, 25092, 25093 | EE0800, E0801, E0810, E0811, E0821, E0822, E0829, E08311, E08319 , E083211 , E083212 , E083213 , E083219 , E083291 , E083292 , E083293 , E083299 , E083311 , E083312 , E083313 , E083319 , E083391 , E083392 , E083393 , E083399 , E083411 , E083412 , E083413 , E083419 , E083491 , E083492 , E083493 , E083499 , E083511 , E083512 , E083513 , E083519 , E083521 , E083522 , E083523 , E083529 , E083531 , E083532 , E083533 , E083539 , E083541 , E083542 , E083543 , E083549 , E083551 , E083552 , E083553 , E083559 , E083591 , E083592 , E083593 , E083599 , E0836 , E0837X1 , E0837X2 , E0837X3 , E0837X9 , E0839 , E0840 , E0841 , E0842 , E0843 , E0844 , E0849 , E0851 , E0852 , E0859 , E08610 , E08618 , E08620 , E08621 , E08622 , E08628 , E08630 , E08638 , E08641 , E08649 , E0865 , E0869 , E088 , E089 , E0900 , E0901 , E0910 , E0911 , E0921 , E0922 , E0929 , E09311 , E09319 , E093211 , E093212 , E093213 , E093219 , E093291 , E093292 , E093293 , E093299 , E093311 , E093312 , E093313 , E093319 , E093391 , E093392 , E093393 , E093399 , E093411 , E093412 , E093413 , E093419 , E093491 , E093492 , E093493 , E093499 , E093511 , E093512 , E093513 , E093519 , E093521 , E093522 , E093523 , E093529 , E093531 , E093532 , E093533 , E093539 , E093541 , E093542 , E093543 , E093549 , E093551 , E093552 , E093553 , E093559 , E093591 , E093592 , E093593 , E093599 , E0936 , E0937X1 , E0937X2 , E0937X3 , E0937X9 , E0939 , E0940 , E0941 , E0942 , E0943 , E0944 , E0949 , E0951 , E0952 , E0959 , E09610 , E09618 , E09620 , E09621 , E09622 , E09628 , E09630 , E09638 , E09641 , E09649 , E0965 , E0969 , E098 , E099 , E1010 , E1011 , E1021 , E1022 , E1029 , E10311 , E10319 , E103211 , E103212 , E103213 , E103219 , E103291 , E103292 , E103293 , E103299 , E103311 , E103312 , E103313 , E103319 , E103391 , E103392 , E103393 , E103399 , E103411 , E103412 , E103413 , E103419 , E103491 , E103492 , E103493 , E103499 , E103511 , E103512 , E103513 , E103519 , E103521 , E103522 , E103523 , E103529 , E103531 , E103532 , E103533 , E103539 , E103541 , E103542 , E103543 , E103549 , E103551 , E103552 , E103553 , E103559 , E103591 , E103592 , E103593 , E103599 , E1036 , E1037X1 , E1037X2 , E1037X3 , E1037X9 , E1039 , E1040 , E1041 , E1042 , E1043 , E1044 , E1049 , E1051 , E1052 , E1059 , E10610 , E10618 , E10620 , E10621 , E10622 , E10628 , E10630 , E10638 , E10641 , E10649 , E1065 , E1069 , E108 , E109 , E1100 , E1101 , E1110 , E1111 , E1121 , E1122 , E1129 , E11311 , E11319 , E113211 , E113212 , E113213 , E113219 , E113291 , E113292 , E113293 , E113299 , E113311 , E113312 , E113313 , E113319 , E113391 , E113392 , E113393 , E113399 , E113411 , E113412 , E113413 , E113419 , E113491 , E113492 , E113493 , E113499 , E113511 , E113512 , E113513 , E113519 , E113521 , E113522 , E113523 , E113529 , E113531 , E113532 , E113533 , E113539 , E113541 , E113542 , E113543 , E113549 , E113551 , E113552 , E113553 , E113559 , E113591 , E113592 , E113593 , E113599 , E1136 , E1137X1 , E1137X2 , E1137X3 , E1137X9 , E1139 , E1140 , E1141 , E1142 , E1143 , E1144 , E1149 , E1151 , E1152 , E1159 , E11610 , E11618 , E11620 , E11621 , E11622 , E11628 , E11630 , E11638 , E11641 , E11649 , E1165 , E1169 , E118 , E119 , E1300 , E1301 , E1310 , E1311 , E1321 , E1322 , E1329 , E13311 , E13319 , E133211 , E133212 , E133213 , E133219 , E133291 , E133292 , E133293 , E133299 , E133311 , E133312 , E133313 , E133319 , E133391 , E133392 , E133393 , E133399 , E133411 , E133412 , E133413 , E133419 , E133491 , E133492 , E133493 , E133499 , E133511 , E133512 , E133513 , E133519 , E133521 , E133522 , E133523 , E133529 , E133531 , E133532 , E133533 , E133539 , E133541 , E133542 , E133543 , E133549 , E133551 , E133552 , E133553 , E133559 , E133591 , E133592 , E133593 , E133599 , E1336 , E1337X1 , E1337X2 , E1337X3 , E1337X9 , E1339 , E1340 , E1341 , E1342 , E1343 , E1344 , E1349 , E1351 , E1352 , E1359 , E13610 , E13618 , E13620 , E13621 , E13622 , E13628 , E13630 , E13638 , E13641 , E13649 , E1365 , E1369 , E138 , E139 , O24011 , O24012 , O24013 , O2402 , O2403 , O24111 , O24112 , O24113 , O2412 , O2413 , O24311 , O24312 , O24313 , O2432 , O2433 , O24811 , O24812 , O24813 , O2482 , O2483 , O24911 , O24912 , O24913 , O2492 , O2493 |
| Gestational Hypertension | 642.3x (without preeclampsia/eclampsia or pre-existing hypertension) | O131, O132, O133, O134, O135, O139 |
| Mild Pre-Eclampsia or Unspecified Pre-Eclampsia | 642.4x, 642.7x (without severe preeclampsia/eclampsia) | O140, O1400, O1402, O1403, O1404, O1405, O149, O1490, O1492, O1493, O1494, O1495 |
| Severe Pre-Eclampsia | 642.5x, 642.6x | O1410, O1412, O1413, O1414, O1415, O1420, O1424, O1425, O1500, O1502, O1503, O151, O152, O159 |
| Chronic Hypertension | 401.x–405.x, 642.0x–642.2x, 642.7x | I10, I110, I119, I120, I129, I130, I1310, I1311, I132, I150, I151, I152, I158, I159, I160, I161, I169, N262, O10011, O10012, O10013, O10019, O1002, O1003, O10111, O10112, O10113, O10119, O1012, O1013, O10211, O10212, O10213, O10219, O1022, O1023, O10311, O10312, O10313, O10319, O1032, O1033, O10411, O10412, O10413, O10419, O1042, O1043, O10911, O10912, O10913, O10919, O1092, O1093, O111, O112, O113, O114, O115, O119 |
| Asthma | 493, 4930, 49300, 49301, 49302, 4931, 49311, 49312, 4932, 49320, 4938, 49381, 49382, 4939, 49390, 49391, 49392 | J440, J441, J449, J4520, J4521, J4522, J4530,, J4531, J4532,, J4540, J4541, J4542, J4550, J4551, J4552, J45901, J45902, J45909, J45991, J45998 |
| Multiple Gestation | V272 , V273 , V274 , V275 , V276 , V277 , 651 , 6510 , 65100, 65101, 65103, 6511 , 65110, 65111, 65113, 6512 , 65120, 65121, 65123, 6513 , 65130, 65131, 65133, 6514 , 65140, 65141, 65143, 6515 , 65150, 65151, 65153, 6516 , 65160, 65161, 65163, 6517 , 65170, 65171, 65173, 6518 , 65180, 65181, 65183, 6519 , 65190, 65191, 65193, 6526 , 65260, 65261, 65263, 6623 , 66230, 66231, 66233, 6605 , 66050, 66051, 66053 | O30001, O30002, O30003, O30009, O30011, O30012, O30013, O30019, O30031, O30032, O30033, O30039, O30041, O30042, O30043, O30049, O30091, O30092, O30093, O30099, O30101, O30102, O30103, O30109, O30111, O30112, O30113, O30119, O30121, O30122, O30123, O30129, O30191, O30192, O30193, O30199, O30201, O30202, O30203, O30209, O30211, O30212, O30213, O30219, O30221, O30222, O30223, O30229, O30291, O30292, O30293, O30299, O30801, O30802, O30803, O30809, O30811, O30812, O30813, O30819, O30821, O30822, O30823, O30829, O30891, O30892, O30893, O30899, O3090, O3091, O3092, O3093, O3110X0, O3110X1, O3110X2, O3110X3, O3110X4, O3110X5, O3110X9, O3111X0, O3111X1, O3111X2, O3111X3, O3111X4, O3111X5, O3111X9, O3112X0, O3112X1, O3112X2, O3112X3, O3112X4, O3112X5, O3112X9, O3113X0, O3113X1, O3113X2, O3113X3, O3113X4, O3113X5, O3113X9, O3120X0, O3120X1, O3120X2, O3120X3, O3120X4, O3120X5, O3120X9, O3121X0, O3121X1, O3121X2, O3121X3, O3121X4, O3121X5, O3121X9, O3122X0, O3122X1, O3122X2, O3122X3, O3122X4, O3122X5, O3122X9, O3123X0, O3123X1, O3123X2, O3123X3, O3123X4, O3123X5, O3123X9, O3130X0, O3130X1, O3130X2, O3130X3, O3130X4, O3130X5, O3130X9, O3131X0, O3131X1, O3131X2, O3131X3, O3131X4, O3131X5, O3131X9, O3132X0, O3132X1, O3132X2, O3132X3, O3132X4, O3132X5, O3132X9, O3133X0, O3133X1, O3133X2, O3133X3, O3133X4, O3133X5, O3133X9, O318X10, O318X11, O318X12, O318X13, O318X14, O318X15, O318X19, O318X20, O318X21, O318X22, O318X23, O318X24, O318X25, O318X29, O318X30, O318X31, O318X32, O318X33, O318X34, O318X35, O318X39, O318X90, O318X91, O318X92, O318X93, O318X94, O318X95, O318X99, O329XX0, O329XX1, O329XX2, O329XX3, O329XX4, O329XX5, O329XX9, O632, O661, O666, Z372, Z373, Z374, Z3750, Z3751, Z3752, Z3753, Z3754, Z3759, Z3760, Z3761, Z3762, Z3763, Z3764, Z3769, Z377 |
| Transfusion | 990, 9900, 9901, 9902, 9903, 9904, 9905, 9906, 9907, 9908, 9909 | 30233H1, 30233K1, 30233L1, 30233M1, 30233N1, 30233P1, 30233R1, 30233T1, 30240H1, 30240K1, 30240L1, 30240M1, 30240N1, 30240P1, 30240R1, 30240T1, 30243H1, 30243K1, 30243L1, 30243M1, 30243N1, 30243N1, 30243P1, 30243R1, 30243T1, 30233N0, 30233P0, 30240N0, 30240P0, 30243N0, 30243P0 |
| Antenatal Hemorrhage | 6413 , 64130, 64131, 64133, 6418 , 64180, 64181, 64183, 6419 , 64190, 64191, 64193 | O45001, O45002, O45003, O45009, O45011, O45012, O45013, O45019, O45021, O45022, O45023, O45029, O45091, O45092, O45093, O45099, O46001, O46002, O46003, O46009, O46011, O46012, O46013, O46019, O46021, O46022, O46023, O46029, O46091, O46092, O46093, O46099, O468X1, O468X2, O468X3, O468X9, O4690, O4691, O4692, O4693, O670, O678, O679 |
| Postpartum Hemorrhage | 66600, 66604, 66610, 66612, 66614, 66620, 66622, 66624, 66630, 66632, 66634 | O720, O721, O722, O723 |
| Placental Abruption | 6412 , 64120, 64121, 64123 | O458X1, O458X2, O458X3, O458X9, O4590, O4591, O4592, O4593 |
| Stillbirth | V271, V273, V276, V277, 6564, 65640, 65641, 65643 | O364XX0, O364XX1, O364XX2, O364XX4, O364XX5, O364XX9, Z371, Z373, Z3760, Z3761, Z3762, Z3763, Z3764, Z3769, Z377 |
| Cesarean Delivery | 74, 740, 741, 742, 744, 749, 7499, 6697, 66970, 66971 | 10D00Z0, 10D00Z1, 10D00Z2 |
| Operative Vaginal Delivery | 66071, 66073, 720, 721, 7221, 7229, 7231, 7239, 7271, 7279 | 10D07Z3, 10D07Z4, 10D07Z5, 10D07Z6, 10D07Z7, 10D07Z8 |
| Prior Cesarean Delivery | 6542, 65420, 65421, 65423 | O3421, O34211, O34212, O34219 |
| Preterm Birth | 6442, 64420, 64421 | O6010X0, O6010X1, O6010X2, O6010X3, O6010X4, O6010X5, O6010X9, O6012X0, O6012X1, O6012X2, O6012X3, O6012X4, O6012X5, O6012X9, O6013X0, O6013X1, O6013X2, O6013X3, O6013X4, O6013X5, O6013X9, O6014X0, O6014X1, O6014X2, O6014X3, O6014X4, O6014X5, O6014X9, O6000, O6002, O6003, O4700, O4702, O4703 |
| Obesity | 278, 2780, 27800, 27801, 27803, 6491, 64910, 64911, 64912, 64913, 64914, 79391, V8530, V853, V8531, V8532, V8533, V8534, V8535, V8536, V8537, V8538, V8539, V8541, V8542, V8543, V8544, V8545, V855 | O91220, O91221, O91222, O91223, O91224, O91225, Z6830, Z6831, Z6832, Z6833, Z6834, Z6835, Z6836, Z6837, Z6838, Z6839, Z6841, Z6842, Z6843, Z6844, Z6845, E660, E6601, E6609, E661, E662, E668, E669 |
| Severe Maternal Morbidity Indicator | | |
| Acute Myocardial Infarction | 410.xx | I21.xx, I22.x |
| Aneurysm | 441.xx | I17.xx, 179.0 |
| Acute Renal Failure | 584.5, 584.6, 584.7, 584.8, 584.9, 669.3x | N17.x, O90.4 |
| Acute Respiratory Distress Syndrome | 518.5x, 518.81, 518.82, 518.84, 799.1 | J80, J95.1, J95.2, J95.3, J95.82x, J96.0x, J96.2x, J96.9x, R06.03, R09.2 |
| Amniotic Fluid Embolism | 673.1x | O88.112, O88.113, O88.119, O88.12, O88.13 |
| Cardiac Arrest/Ventricular Fibrillation | 427.41, 427.42, 427.5 | I46.x, I49.0x |
| Conversion of Cardiac Rhythm | 99.6x | 5A12012, 5A2204Z |
| Disseminated Intravascular Coagulation | 286.6, 286.9, 641.3x, 666.3x | D65, D68.8, D68.9, O45.002, O45.003, O45.009, O45.012, O45.013, O45.019, O45.022, O45.023, O45.029, O45.092, O45.093, O45.099, O46.002, O46.003, O46.009, O46.012, O46.013, O46.019, O46.022, O46.023, O46.029, O46.092, O46.093, O46.099, O67.0, O72.3 |
| Eclampsia | 642.6x | O15.X |
| Heart Failure/Arrest During Surgery | 997.1 | I97.120, I97.121, I97.130, I97.131, I97.710, I97.711 |
| Puerperal Cerebrovascular Disorders | 046.3, 348.39, 362.34, 430.xx, 431.xx, 432.xx, 433.xx, 434.xx, 435.xx, 436.xx, 437.xx, 671.5x, 674.0x, 997.02 | A81.2, G45.x, G46.x, G93.49, H34.0x, I60.xx, I61.xx, I62.xx, I63.00, I63.01x, I63.1xx, I63.2xx, I63.3xx, I63.4xx, I63.5xx, I63.6, I63.8x, I63.9, I65.xx, I66.xx, I67.xx, I68.xx, O22.50, O22.52, O22.53, I97.810, I97.811, I97.820, I97.821, O87.3 |
| Pulmonary Edema/Acute Heart Failure | 428.0, 428.1, 428.20, 428.21, 428.23, 428.30, 428.31, 428.33, 428.40, 428.41, 428.43, 428.9, 518.4 | I50.1, I50.20, I50.21, I50.23, I50.30, I50.31, I50.33, I50.40, I50.41, I50.43, I50.810, I50.811, I50.813, I50.814, I50.82, I50.83, I50.84, I50.89, I50.9, J81.0 |
| Severe Anesthesia Complication | 668.0x, 668.1x, 668.2x, 995.4, 995.86 | O29.112–O29.119, O29.122–O29.129, O29.192–O29.199, O29.212–O29.219, O29.292–O29.299, O74.0, O74.1, O74.2, O74.3, O89.0x, O89.1, O89.2, T88.2XXA, T88.3XXA |
| Sepsis | 038.xx, 449, 785.52, 995.91, 995.92, 998.02, 670.2x (after October 1, 2009) | A32.7, A40.x, A41.x, I76, O85, O86.04, R65.20, R65.21, T81.12XA, T81.44XA |
| Shock | 669.1x, 785.50, 785.51, 785.59, 995.0, 998.0*, 998.00, 998.01, 998.09 *998.0 is not a valid code but was used prior to 2012 | O75.1, R57.x, T78.2XXA, T81.10XA, T81.11XA, T81.19XA, T88.6XXA |
| Sickle Cell Disease with Crisis | 282.42, 282.62, 282.64, 282.69, 289.52 | D57.00, D57.01, D57.02, D57.211, D57.212, D57.219, D57.411, D57.412, D57.419, D57.811, D57.812, D57.819 |
| Air and Thrombotic Embolism | 415.0, 415.1x, 673.0x, 673.2x, 673.3x, 673.8x | I26.x, O88.012–O88.03, O88.212–O88.23, O88.312–O88.33, O88.812–O88.83, T80.0XXA |
| Hysterectomy | 68.39, 68.49, 68.59, 68.69, 68.79, 68.9 (also include 68.3, 68.4, 68.5, 68.6, 68.7; non-specific codes used frequently) | 0UT90ZL, 0UT90ZZ, 0UT97ZL, 0UT97ZZ |
| Temporary Tracheostomy | 31.1 | 0B110F4, 0B113F4, 0B114F4 |
| Ventilation | 96.70, 96.71, 96.72 | 5A1935Z, 5A1945Z, 5A1955Z |
