# Supplementary material for: Contemporary trends in maternal outcomes during delivery hospitalizations among pregnancies complicated by von Willebrand disease—a cross-sectional analysis
Source: Res Pract Thromb Haemost. 2025 Sep 5;9(6):103174. doi: 10.1016/j.rpth.2025.103174 (PMC12509092; doi:10.1016/j.rpth.2025.103174)
Supplement: Supplementary Table 2 [file mmc2.docx]

| **Supplemental Table 2: Multivariable Logistic Regression of Outcomes Associated with von Willebrand Disease**  *The adjusted model included demographic factors (maternal age, maternal race, payer status, and median income quartile), clinical factors (chronic hypertension, pre-gestational diabetes, prior cesarean delivery, multifetal gestation, obesity, and asthma), hospital factors (teaching hospital status and region), and delivery year. | | | | |
| --- | --- | --- | --- | --- |
| **Outcome** | **Unadjusted** | | **Adjusted*** | |
|  | **OR** | **95% CI** | **aOR** | **95% CI** |
| Hypertensive Disorders of Pregnancy | 1.29 | 1.20, 1.39 | 1.09 | 1.01, 1.18 |
| Placental Abruption/Antepartum Hemorrhage | 1.83 | 1.56, 2.13 | 1.81 | 1.55, 2.12 |
| Postpartum Hemorrhage | 1.79 | 1.63, 1.98 | 1.68 | 1.52, 1.85 |
| Transfusion | 5.00 | 4.46, 5.60 | 5.12 | 4.55, 5.75 |
| Thrombotic Complication | 1.73 | 0.98, 3.05 | 1.51 | 0.86, 2.67 |
| Non-transfusion Severe Maternal Morbidity | 2.63 | 2.22, 3.12 | 2.54 | 2.14, 3.01 |
| Cesarean Delivery | 1.17 | 1.11, 1.23 | 1.16 | 1.09, 1.22 |
| Operative Vaginal Delivery | 0.71 | 0.63, 0.80 | 0.82 | 0.61, 1.11 |
| Stillbirth | 0.87 | 0.65, 1.16 | 0.93 | 0.70, 1.25 |
| Preterm Birth <28w | 1.16 | 0.74, 1.80 | 1.14 | 0.73, 1.79 |
| Preterm Birth <32w | 0.91 | 0.66, 1.24 | 0.87 | 0.63, 1.19 |
| Preterm Birth <37w | 1.11 | 0.99, 1.25 | 1.09 | 0.96, 1.23 |
